# Macrolides rapidly inhibit red blood cell invasion by the human malaria parasite, *Plasmodium falciparum*

**DOI:** 10.1186/s12915-015-0162-0

**Published:** 2015-07-18

**Authors:** Danny W Wilson, Christopher D Goodman, Brad E Sleebs, Greta E Weiss, Nienke WM de Jong, Fiona Angrisano, Christine Langer, Jake Baum, Brendan S Crabb, Paul R Gilson, Geoffrey I McFadden, James G Beeson

**Affiliations:** Research Centre for Infectious Diseases, School of Biological Sciences, The University of Adelaide, Adelaide, South Australia; Walter and Eliza Hall Institute of Medical Research, Parkville, Victoria 3050 Australia; Department of Medical Biology, University of Melbourne, Parkville, Victoria 3050 Australia; Macfarlane Burnet Institute for Medical Research and Public Health, Melbourne, Victoria 3004 Australia; Plant Cell Biology Research Centre, School of Biosciences, University of Melbourne, Parkville, Victoria 3010 Australia; Department of Life Sciences, Imperial College London, South Kensington, London, SW7 2AZ UK; Department of Immunology, Monash University, Clayton, Victoria 3800 Australia; Department of Microbiology, Monash University, Clayton, Victoria 3168 Australia

**Keywords:** *Plasmodium*, Merozoite, Invasion, Macrolide, Dual modality

## Abstract

**Background:**

Malaria invasion of red blood cells involves multiple parasite-specific targets that are easily accessible to inhibitory compounds, making it an attractive target for antimalarial development. However, no current antimalarial agents act against host cell invasion.

**Results:**

Here, we demonstrate that the clinically used macrolide antibiotic azithromycin, which is known to kill human malaria asexual blood-stage parasites by blocking protein synthesis in their apicoplast, is also a rapid inhibitor of red blood cell invasion in human (*Plasmodium falciparum*) and rodent (*P. berghei*) malarias. Multiple lines of evidence demonstrate that the action of azithromycin in inhibiting parasite invasion of red blood cells is independent of its inhibition of protein synthesis in the parasite apicoplast, opening up a new strategy to develop a single drug with multiple parasite targets. We identified derivatives of azithromycin and erythromycin that are better invasion inhibitors than parent compounds, offering promise for development of this novel antimalarial strategy.

**Conclusions:**

Safe and effective macrolide antibiotics with dual modalities could be developed to combat malaria and reduce the parasite’s options for resistance.

**Electronic supplementary material:**

The online version of this article (doi:10.1186/s12915-015-0162-0) contains supplementary material, which is available to authorized users.

## Background

Malaria is a mosquito-borne parasitic disease, which causes nearly 600,000 deaths every year, mainly in children under 5 years of age in sub-Saharan Africa [1]. Most deaths are attributed to the most virulent human malaria parasite, *Plasmodium falciparum*. Current disease control measures rely on reducing exposure to the mosquito vector or treatment of clinical malaria cases with antimalarial drugs that target the disease causing blood-stage trophozoite; most of these act against the parasite’s food vacuole or interfere with pyrimidine synthesis of maturing trophozoites [2–6]. Of major concern is the emerging development of drug resistance to the highly effective artemisinin family of compounds in Southeast Asia [7–9]. The prospect of widespread resistance to the most effective antimalarials becoming entrenched has led to the implementation of an emergency response plan by the World Health Organization (WHO) to prevent the spread of resistance and the inevitable increase in childhood mortality that would result [10]. This developing emergency highlights the urgent need for new antimalarials with novel mechanisms of action for use in combination with artemisinins, or to replace these drugs in the future.

Invasion of blood-stage merozoites into host cells has been proposed as a potential target for antimalarial chemotherapy [11–14]. Invasion of the extracellular merozoite into a new host erythrocyte (red blood cell) is a complex process requiring the coordinated interaction of unique parasite ligands, signaling pathways, and a form of gliding motility powered by an actomyosin motor. The multiple accessible targets make this essential stage of the lifecycle an attractive drug target. In addition, inhibition of merozoite invasion into the erythrocyte would lead to instant disruption of the parasite lifecycle and prevent sequestration, dormancy and commitment to the mosquito-transmissible gametocyte stage, which have been reported to occur after drug treatment with current first-line antimalarials [15–17]. Application of antimalarial drugs that target invasion has the potential to result in quicker resolution of clinical disease if used in combination with antimalarials that act at other developmental stages.

Few compounds have been directly tested for ability to inhibit *P. falciparum* merozoite invasion, partly through lack of tractable invasion inhibition assays. The ability to assess the invasion inhibitory potential of compounds and their biological activity *in vitro* has been greatly enhanced with the development of a robust and efficient merozoite purification protocol and techniques to directly measure invasion inhibition [13, 18–20], allowing the accurate and rapid assessment of a compounds invasion inhibitory potential. While some invasion inhibitory compounds (e.g. protease inhibitors [18, 21, 22], heparin [23] and calcium signaling modulators [23, 24]) have been reported, to date no drug used clinically to treat malaria has been shown to have inhibitory activity against merozoite invasion [13]. In this study, we have identified clinically relevant macrolide antibiotics and related compounds, exemplified by azithromycin, as rapid inhibitors of *P. falciparum* and *P. berghei* merozoite invasion *in vitro*. This represents the first time that a drug or drug class that has clinical applications against malaria has been demonstrated to inhibit parasite invasion of the red blood cell.

Antibiotics such as azithromycin and clindamycin (lincosamide antibiotic) have been proposed as potential partner drugs for artemisinin combination therapies owing to their extremely long half-life and good safety profile *in vivo* [25–29]. Both azithromycin and clindamycin inhibit apicoplast ribosomal protein synthesis of asexual blood-stage parasites at nanomolar (clinical) concentrations by binding to the apicoplast ribosomal 50S subunit and blocking protein exit from the ribosome [30–32]. *In vitro* experiments at clinically relevant concentrations show that these antibiotics have a ‘delayed death’ drug response. Treated parasites grow normally during the first lifecycle under treatment. Only during the second post-treatment cycle (after replication and reinvasion) are drug effects observed. Parasite death at this stage is thought to result from the inheritance of a defective apicoplast that is unable to synthesize isoprenoid precursors required for development [31–33]. This ‘delayed death’ response currently limits the usefulness of azithromycin and clindamycin for the treatment of clinical disease as stand-alone drugs, and successful combinations with other antimalarials remain elusive [34].

In this study, we aimed to identify existing, and novel, compounds with invasion inhibitory activity that have the potential for development as effective antimalarials. We demonstrate that the antibiotic azithromycin can specifically inhibit merozoite invasion. Related macrolide antibiotics were also found to inhibit invasion, and the IC_50_ of invasion inhibitory activity could be lowered through modification. These results provide the basis for a novel strategy of antimalarial drug development by advancing compounds that have dual mechanisms of action. Given the established safety and low cost of macrolides in widespread clinical use, development of macrolides as antimalarials with dual modalities against merozoite invasion and protein translation in the apicoplast is a promising strategy to counter the rapid emergence of drug resistance.

## Results

### Azithromycin inhibits merozoite invasion

**Fig. 1:**
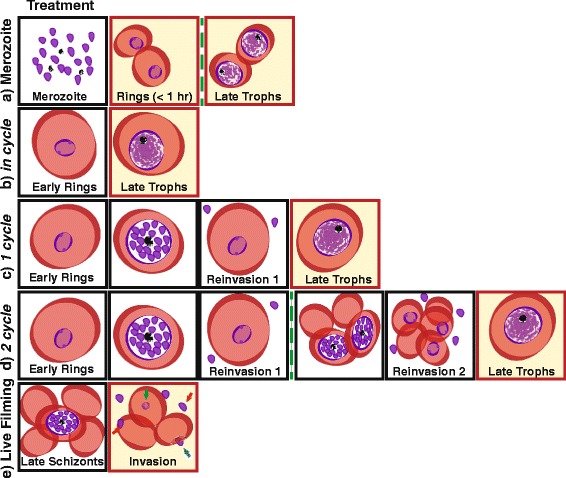
Drug treatment strategies used in this study. The lifecycle stage of drug treatment is represented in the first box for each panel and the stage of parasitaemia measurements are highlighted by red boxes with yellow background. (**a**) Merozoite; successful invasion of drug-treated purified merozoites was measured at ring-stage immediately after addition of erythrocytes and merozoite invasion (<1 hour rings) or after washing out the drug (denoted by green dashed line) and growing parasites through to late trophozoite stage (40 hours post-invasion). (**b**) In cycle; early ring-stage parasites (<4 hours post-invasion) were drug-treated and the resulting growth inhibition was assessed at late trophozoite stage (40 hours post-invasion). (**c**) 1 cycle; early ring-stage parasites (<4 hours post-invasion) were drug-treated and the resulting growth inhibition was assessed 90 hours later, after 1 cycle of reinvasion, at late trophozoite stage. These assays are a longer duration, but nonetheless equivalent in terms of including 1 cycle of reinvasion and development, to 1 cycle assays reported in other studies [41–44]. (**d**) 2 cycle (delayed death); early ring-stage parasites (<4 hours post-invasion) were drug-treated and the drug-treated parasites were grown for 80 hours prior to washing out the drug in fresh media (denoted by green dashed line). Growth inhibition was assessed approximately 40 hours later after a second cycle of reinvasion (120 hours post-invasion). (**e**) Live filming; mature schizont-stage parasites were incubated with azithromycin and the success of merozoite invasion was recorded and quantified by live filming (green arrow, successful invasion; blue arrow, deformation of the erythrocyte but no invasion; red arrow, attachment and release or attachment with failure to deform erythrocyte and no release) (see Additional file 1: Video S1 and Additional file 2: Video S2). White box-drug treatment; yellow box-analysis of parasitaemia; green dashed line-drug washout

**Fig. 2:**
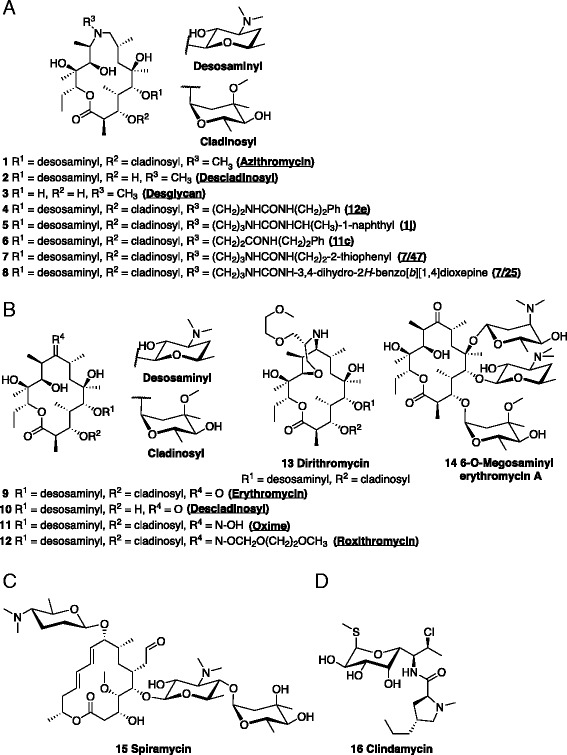
Structure of macrolide antibiotics. (**a**) Structure of the 15-membered macrolide, azithromycin, and its modified analogues (names used in the text underlined, in brackets). (**b**) Structure of the 14-membered macrolide, erythromycin A, and its modified analogues. (**c**) Structure of the 16-membered macrolide, spiramycin. (**d**) Structure of the non-macrolide antibiotic, clindamycin

**Fig. 3:**
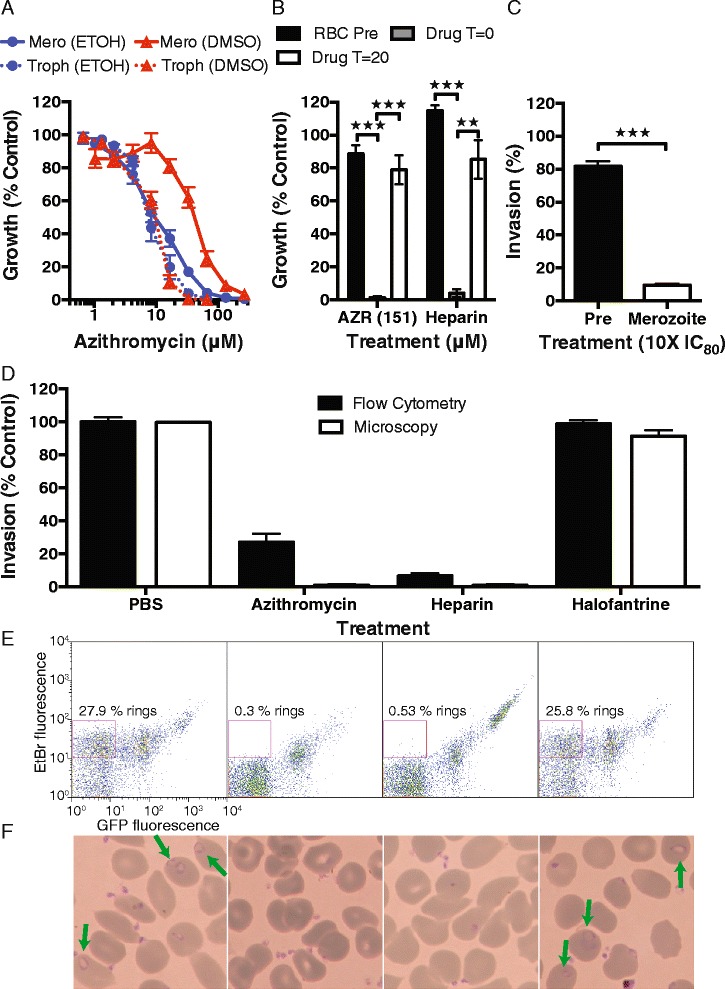
Azithromycin inhibits *P. falciparum* merozoite invasion. (**a**) The potency of azithromycin in ethanol or DMSO as vehicle was compared for invasion inhibition (unbroken line, 10 minute merozoite treatment, parasitaemia measured 40 hours later) and 1 cycle growth inhibition assays (broken line, treatment rings to trophozoites’ next cycle). The invasion inhibitory IC_50_ of azithromycin prepared in ethanol (blue, IC_50_ 10 μM) was similar to that for growth inhibition assays (IC_50_ 7 μM; *P* = 0.0743, Log IC_50_ same between data sets, extra sum of squares F-test). The invasion inhibitory activity of azithromycin in DMSO (red, IC_50_ 38 μM) was 5-fold higher than 1 cycle growth assays (IC_50_ 7 μM; *P* <0.0001, Log IC_50_ different between data sets). (**b**) Inhibition profiles for pretreated erythrocytes (RBC Pre), merozoite treatment (T = 0; drug added at time zero) and rings treated for <1 hour (T = 20; drug added 20 minutes post-invasion) were identical between azithromycin (in DMSO) and the invasion inhibitor heparin (IC_80_ concentration). (**c**) Increasing the concentration of azithromycin (in ethanol) to 10 × IC_80_ (380 μM) did not result in substantial inhibition of invasion into pretreated cells compared to treatment of merozoites. (**d**) Flow cytometry and microscopy assessments confirmed that azithromycin (IC_80_ in ethanol) and heparin, but not the trophozoite-targeting antimalarial halofantrine (2 × IC_80_ ring-stage treatment (46 nM) [13]), inhibit merozoite invasion and establishment of ring stages in erythrocytes. Representative (**e**) flow cytometry plots (GFP high and EtBr low ring-stage parasites represented by square gate) and (**f**) microscopy thin smears (rings highlighted by green arrows) show absence of ring-stage parasites for azithromycin and heparin compared to non-invasion inhibitory controls (labeling as per Fig. 3d). Experiments represent the mean and SEM of three or more experiments. Significance was tested using an unpaired t-test (*P = 0.01–0.05, **P ≤0.01, ***P ≤0.001)

Application of the merozoite purification method of Boyle et al. [18] identified the macrolide antibiotic azithromycin as a candidate inhibitor of *P. falciparum* asexual blood-stage merozoite invasion of the host erythrocyte (see Fig. 1a for representation of assay setup, Fig. 2 for structure of azithromycin and other drugs used in this study). Initial screens indicated that the invasion inhibitory IC_50_ differed between azithromycin prepared in ethanol (10 μM) or DMSO (38 μM), suggesting that choice of vehicle can impact azithromycin potency *in vitro*. Comparative screens of azithromycin potency over different treatment times demonstrated that the IC_50_ of merozoite invasion assays (1 hour exposure to drug) were surprisingly similar to the in cycle (40 hour incubation, no invasion step, Fig. 1b) and 1 cycle (90 hour, one invasion step, Fig. 1c) assays (IC_50_ –(drug prepared in ethanol) invasion 10 μm; in cycle 6 μM; 1 cycle 7 μM, −(drug prepared in DMSO) invasion 38 μM (DMSO); in cycle 12 μM; 1 cycle assay 7 μM, Fig. 3a, Table 1). These results suggest that azithromycin can act independently against both merozoite invasion and intracellular parasite growth within one cycle of treatment *in vitro*.

**Table 1:** Growth/invasion inhibitory activity of compounds described in this study

Compound number	Compound abbreviation	Compound name	Lactone ring size	IIA IC_50_ (μM)	40 hour IC_50_ (μM)	90 hour IC_50_ (μM)	120 hour IC_50_ (μM)
1	AZR^a^	Azithromycin	15	10	6	7	0.04
2	AZR-desclad^a^	-	15	50	39	31	ND
3	AZR-desglycan^a^	-	15	>1,600	>500	285	ND
1	AZR^b^	Azithromycin	15	38	12	7	0.1
4	12e^b,c^	-	15	15	1	0.6	0.16
5	1j^b,c^	-	15	7	0.9	0.7	0.16
6	11c^b,d^	-	15	28	ND	0.9	ND
7	7/47^b,e^	-	15	52	ND	5	ND
8	7/25^b,e^	-	15	73	ND	10	ND
9	ERY^a^	Erythromycin	14	420	230	52	ND
10	ERY-desclad^a^	-	14	ND	288	185	ND
11	ERY-oxime^a^	-	14	150	85	24	ND
12	ROX^a^	Roxithromycin	14	83	70	27	ND
13	DIR^a^	Dirithromycin	14	521	15	8	ND
14	Meg-ERY^a,f^	6-0-megosaminyl-erythromycin	14	13	ND	ND	ND
15	SPI^a^	Spiramycin	16	123	15	13	ND
16	CLI^a^	Clindamycin	NA	743	ND	ND	ND
D10-AZR^r^ (delayed death, resistant) comparison							
1	AZR^a^	Azithromycin	15	-	-	-	0.16^g,h^
1^g^	AZR^a^	Azithromycin	15	25	ND	ND	9^h^

^a^Vehicle was ethanol; ^b^vehicle was dimethyl sulfoxide (DMSO); ^c^from Bukvic et al. [44]; ^d^from Peric et al. [42]; ^e^from Hutinec et al. [43]; ^f^from Goodman et al. [41]. All IC_50_s are for the D10-PfPHG line used throughout this study, with the exception of ^g^which were undertaken with the D10 azithromycin-resistant line (D10-AZR^r^) made as per Goodman et al. [41]. All assays were measured by flow cytometry, with the exception of ^h^which were measured by hypoxanthine uptake assays. Desclad, descladinosyl; desglycan, descladinosyl and desosaminyl; NA, not applicable; ND, not done

To confirm that azithromycin was inhibiting merozoite invasion directly in our assays and was not acting downstream of invasion during intracellular parasite growth, the effect of exposure of merozoites or early ring-stage parasites to the drug was examined; azithromycin was washed out of the cultures within 1 hour and parasitaemia assessed by flow cytometry 40 hours post-assay setup for all treatments (prior to the next round of parasite rupture and invasion of new host cells). Treatment of merozoites (10 minute incubation) and early ring stages for less than 1 hour (T = 0) with azithromycin (1 × IC_80_^*mero*^ DMSO 151 μM) and the invasion inhibitory control compound, heparin, was significantly more inhibitory to parasite growth than the same treatment of early ring stages at the time-point of 20 minutes post-invasion (T = 20 (exposed for 40 minutes); azithromycin *P* <0.001; heparin *P* <0.01). Additionally, pretreatment of erythrocytes, followed by washout of drug, had little or no inhibitory effect on invasion (Fig. 3b; azithromycin *P* <0.001; heparin *P* <0.001). This indicated that exposure of merozoites, but not very early ring stages or uninfected erythrocytes, was inhibitory to parasite growth.

In order to confirm that the inhibitory activity was against purified merozoites and not the uninfected erythrocyte prior to merozoite invasion, erythrocytes were pretreated with azithromycin at a 10 × IC_80_^*mero*^ (380 μM (drug prepared in ethanol to limit nonspecific effects of vehicle)) prior to washing and addition of merozoites. There was minimal loss of invasion into erythrocytes pretreated with a 10 × IC_80_^*mero*^ concentration of azithromycin for 1 hour relative to control (Fig. 3c). In contrast, merozoites treated for 10 minutes with the same concentration of azithromycin were unable to establish erythrocyte infections (*P* <0.001), supporting the conclusion that it is the merozoite and its interaction with the erythrocyte, and not the erythrocyte itself, that is the target of inhibition.

Next we confirmed that azithromycin was inhibiting merozoite invasion directly, rather than acting on growth downstream of invasion, through the complementary methods of microscopy and flow cytometric quantification of ring-stage parasites at the time-point of 1 hour post-invasion. There was an almost complete loss of ring-stage parasites seen by both microscopy and flow cytometry for azithromycin (1 × IC_80_^*mero*^), confirming that azithromycin is indeed inhibitory to merozoite invasion (Fig. 3d,e,f). The merozoite invasion inhibitory activity of azithromycin was unaffected by limiting drug exposure to <5 seconds prior to erythrocyte addition, by the presence of serum in the culture medium or the presence of haemazoin crystals (that can remain after purification of merozoites) in the invasion assay. These results demonstrate that azithromycin is a rapid inhibitor of merozoite invasion *in vitro*.

### Azithromycin inhibits merozoite invasion prior to tight junction formation

**Fig. 4:**
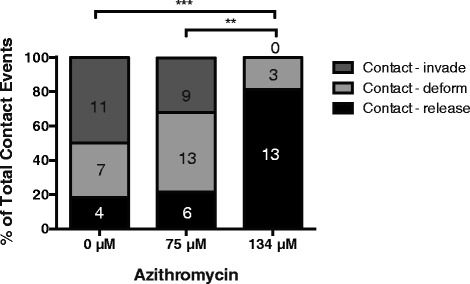
Azithromycin inhibits the early steps of invasion. Video microscopy of merozoite invasion of erythrocytes was performed in the presence of 75 and 134 μM azithromycin (in ethanol) compared to a no drug control (0 μM). Five schizont ruptures were observed for each treatment. Of the merozoites that contacted erythrocytes, some were observed to deform erythrocytes and then successfully invade their host cells (contact–invade), while others did not progress beyond initial attachment (contact–detach) or progressed to deformation but did not invade (contact–deform). From several rupturing schizonts, the number of merozoites exhibiting each of these steps was counted for each drug treatment and the percentages are shown along with the number of events in the column boxes. A Chi-squared test was performed to indicate significant differences at the following levels (**P ≤0.01, ***P ≤0.001)

Live cell imaging of invading *Plasmodium* merozoites indicates that erythrocyte invasion is a multi-step process that typically takes less than 30 seconds to complete, after contact occurs between the merozoite and the erythrocyte, in primate species and slightly longer in rodent species [35–37]. After contacting its target erythrocyte, the merozoite reorients its apical end onto the erythrocyte surface in a process that deforms the erythrocyte. This reorientation lasts about 10 seconds and is followed by the formation of a ring-like region of close and irreversible contact, called the tight (or moving) junction, between the merozoite apex and the erythrocyte. The merozoite enters the host cell through this junction using the power of its actin-myosin motor. After invasion, the erythrocyte surface reseals and the merozoite differentiates into a ring-stage parasite over several minutes [35]. We used live cell imaging to identify invasion step(s) that are inhibited by azithromycin (Fig. 1e). Late schizont-stage parasites were allowed to rupture in the presence of 75 and 134 μM azithromycin (in ethanol), which represent 2 × IC_80_ and 3.5 × IC_80_ of merozoite invasion inhibition concentrations, respectively. These slightly higher drug concentrations were used to clearly define the inhibitory phenotype. These concentrations were not found to inhibit schizont rupture. Five schizont ruptures were observed for each treatment. In the untreated control, 22 merozoites were observed to contact erythrocytes. After a contact period ranging from a few seconds to 2 minutes, 18 % of the merozoites released or detached. A further 32 % of the merozoites progressed to the erythrocyte deformation stage before releasing over a few minutes, and the remaining 50 % advanced to complete invasion (Fig. 4). Azithromycin treatment at 75 μM changed this profile with fewer merozoites invading (32 %) and a greater proportion failing to progress beyond the initial contact and deformation stages (68 %, Fig. 4). At 134 μM there was a significant and dramatic change in the invasion profile compared to the untreated control and azithromycin treatment at 75 μM with no merozoites invading and most releasing after initial contact (81 %, Fig. 4). The observation that merozoites failed to make sustained contact with erythrocytes in the presence of azithromycin indicates that azithromycin acts early in invasion to prevent tight junction formation [23, 35, 38]. The periods of time that each of the invasion steps took to occur was also measured, but in nearly all cases showed no significant difference between the treatments. Representative video files showing the effects of azithromycin are shown in Additional file 1: Video S1 and Additional file 2: Video S2.

### Macrolides related to azithromycin also inhibit merozoite invasion

**Fig. 5:**
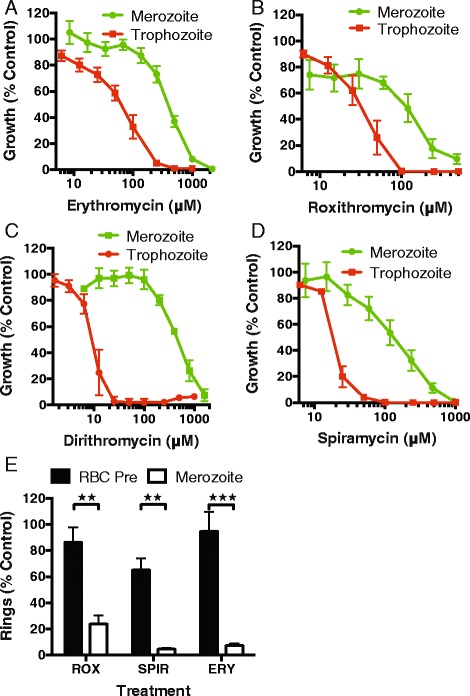
Related macrolides inhibit merozoite invasion. The 14-membered macrolides (**a**) erythromycin A (IC_50_^*mero*^ 420 μM), (**b**) roxithromycin (83 μM), (**c**) dirithromycin (521 μM) and (**d**) the 16-membered macrolide spiramycin (123 μM) had variable levels of invasion inhibitory activity (green) and a higher IC_50_ than that achieved for 1 cycle assays (red). (**e**) The invasion inhibitory activity of erythromycin A, roxithromycin and spiramycin at an IC_80_ concentration was confirmed by flow cytometry assessment of ring stages with minimal inhibition evident for pretreated erythrocytes. All experiments represent the mean and SEM of three or more experiments. Significance of differences was compared using an unpaired t-test (**P ≤0.01, ***P ≤0.001)

After identifying that azithromycin was inhibitory to merozoite invasion, we examined whether related drugs with a history of clinical use as antibiotics also had this property. Macrolides with a 14-membered macrolactone ring (erythromycin A, roxithromycin, dirithromycin) and 16-membered ring (spiramycin) were tested using the same methods as azithromycin (prepared in ethanol, Fig. 2b,c). Determination of the inhibitory concentration (Fig. 5a,b,c,d; Table 1) indicated that azithromycin (ethanol, 10 μM) had >8-fold lower IC_50_ for merozoite invasion when compared to roxithromycin (83 μM) and spiramycin (123 μM), while erythromycin (420 μM) was 42-fold less potent than azithromycin. Of interest was the very poor invasion inhibitory activity of dirithromycin (521 μM), which was 52-fold less potent than azithromycin, even though the IC_50_ values determined using 1 cycle growth assays were very similar (dirithromycin, IC_50_^*90hr*^ 8 μM; azithromycin, IC_50_^*90hr*^ 7 μM; Table 1). Invasion inhibition was confirmed by measurement of ring-stage parasites 1 hour after invasion at 1 × IC_80_^*mero*^ for erythromycin A, roxithromycin and spiramycin. The lack of inhibitory activity when erythrocytes were pretreated with drug (and then washed) further confirmed specific inhibition of merozoite invasion (Fig. 5e; *P* <0.01).

### Merozoite invasion inhibition is independent of macrolide activity against apicoplast ribosomes

**Fig. 6:**
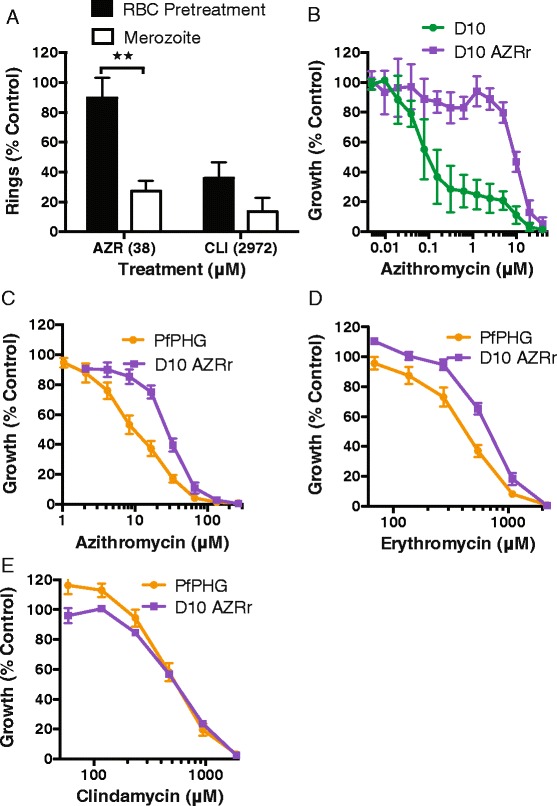
The mechanism of invasion inhibition is unlikely to target the apicoplast ribosome. (**a**) Clindamycin targets the same subunit of the apicoplast ribosome but was found to have a much higher IC_80_ for apparent merozoite invasion inhibition (2,972 μM). There was evidence of non-specific inhibition of invasion as pretreatment of erythrocytes with clindamycin gave significant inhibition, which was not seen for azithromycin (prepared in ethanol; mean and SEM of four or more experiments; significance of differences tested with an unpaired t-test; **P ≤0.01) (38 μM). (**b**) The D10-AZR^r^ line showed up to a 57-fold higher tolerance of azithromycin in 2 cycle (delayed death) apicoplast-targeting drug inhibition assays compared to D10 parental line. In contrast, the IC_50_ for purified merozoite invasion inhibitory activity differed by less than 2.5-fold between the D10-AZR^r^ line and the D10-PfPHG line for (**c**) azithromycin (IC_50_: PfPHG 10 μM; D10-AZR^r^ 25 μM), (**d**) erythromycin A (IC_50_: PfPHG 420 μM; D10-AZR^r^ 732 μM) and (**e**) clindamycin (IC_50_: PfPHG 743 μM; D10-AZR^r^ 557 μM). Data represent the mean of two or more experiments in at least duplicate. D10-AZR^r^, D10 azithromycin-resistant

Since macrolide antibiotics are known to target the 50S ribosomal subunit of the apicoplast to inhibit subsequent intra-erythrocytic development, we tested the invasion inhibitory activity of clindamycin, which has a similar mechanism of action and exhibits overlapping binding of the 50S ribosomal subunit of the apicoplast to azithromycin [39] (Table 1; Fig. 2c). Clindamycin had very weak invasion inhibitory activity; there was evidence for invasion inhibitory activity with a 74-fold higher IC_50_ observed for clindamycin (IC_50_^*mero*^ 743 μM) than seen for azithromycin (10 μM). However, pre-treatment of erythrocytes with 2,972 μM of clindamycin (IC_80_^*mero*^) resulted in a 65 % reduction in merozoite invasion relative to untreated controls and there was evidence of erythrocyte lysis using these high levels of clindamycin (Fig. 6a). This suggests that at such high concentrations clindamycin is having a largely non-specific effect on merozoite invasion through damaging erythrocytes.

We tested further whether apicoplast ribosomal protein translation was the target of the inhibitory activity at the merozoite stage by comparison of the invasion inhibitory activity of azithromycin, erythromycin and clindamycin between the D10-PfPHG (azithromycin-sensitive) parasite used throughout this study and an azithromycin-resistant D10 derivative (D10-AZR^r^) selected for reduced sensitivity to both azithromycin and erythromycin in 2 cycle (delayed death) assays. Sequencing of apicoplast ribosome genes revealed a G91D mutation in the *rpl4* gene product, corresponding to the G112D mutation in *Chlamydomonas reinhardtii*, known to confer resistance to erythromycin [40]. This mutation is associated with a 57-fold loss of sensitivity to azithromycin over 2 cycles of parasite growth (IC_50_^*120hr*^ D10-AZR^r^, 9 μM; D10 parental, 0.16 μM; *P* <0.0001; Fig. 6b; Table 1). Comparison of the IC_50_^*mero*^ (Fig. 6c,d,e) suggested that there was very little difference in merozoite invasion inhibitory activity between the resistant versus sensitive lines for azithromycin (in ethanol, 2.5-fold difference; IC_50_ D10-AZR^r^, 25 μM; D10-PfPHG, 10 μM; *P* <0.0001), erythromycin (1.6-fold difference; IC_50_ D10-AZR^r^, 732 μM; D10-PfPHG, 442 μM; *P* = 0.0247) or clindamycin (1.3-fold difference; IC_50_ D10-AZR^r^, 556 μM; D10-PfPHG, 736 μM; *P* = 0.26). Together, these data suggest that the invasion inhibitory activity of azithromycin is largely independent of apicoplast ribosomal protein synthesis, consistent with the current view that the apicoplast does not play a role in invasion.

### Modification of macrolides enhances invasion inhibitory activity

**Fig. 7:**
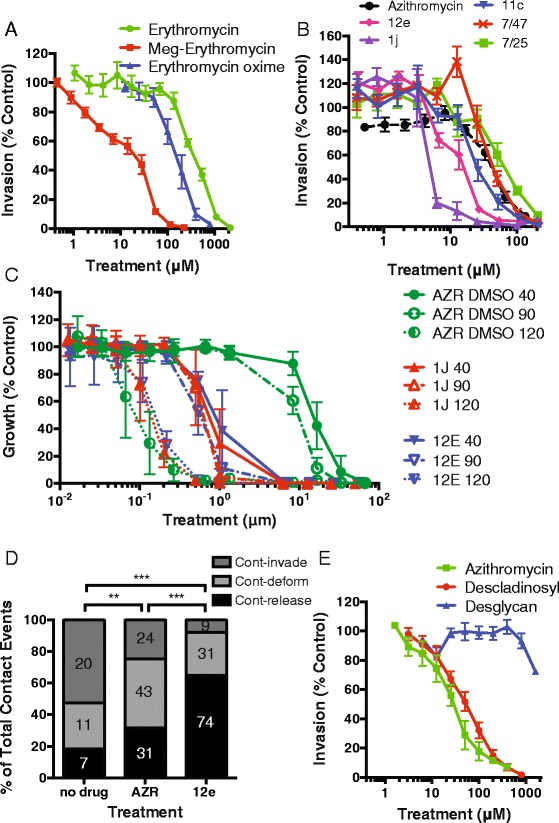
Macrolide modification lowers invasion inhibitory IC_50_, but not apicoplast-targeting ‘delayed death’ inhibition. (**a**) Addition of an L-megosamine sugar [41] to form Meg-erythromycin (6-O-megosaminyl erythromycin A, IC_50_ 13 μM) or an oxime group (150 μM) lowered the invasion inhibitory IC_50_ activity compared to the parent drug erythromycin A (IC_50_ 420 μM). (**b**) Screening of an azithromycin analogue panel identified three compounds (12e, 15 μM; 1j, 7 μM; 11c, 28 μM) with up to 5-fold lower invasion inhibitory IC_50_ compared to the parent azithromycin (in DMSO, 38 μM). (**c**) Treatment of parasites during in cycle (40 hours, rings to schizonts), 1 cycle (90 hours, 1 cycle of replication) and 2 cycle (120 hours, 2 cycles of replication) assays with azithromycin (in DMSO) and analogues (12e, 1j), indicated that the IC_50_ of 1 cycle (40 hour and 90 hour, high drug concentration) inhibition was greatly reduced for the analogues compared to azithromycin. In contrast, the IC_50_ of the delayed death phenotype was almost identical for azithromycin and its analogues. **d**) Video microscopy of merozoite invasion was performed in the presence of a no drug control (0 μM), 122 μM of analogue 12e (2 × IC_80_) and azithromycin (AZR; both in DMSO). Merozoites that contacted the erythrocyte and i) invaded (cont–invade), ii) deformed but did not invade (cont–deform), or iii) released without deforming or invading (cont–detach) were tallied and analyzed as per Fig. 4. (**e**) Removal of the cladinosyl sugar (azithromycin descladinosyl, 50 μM) from azithromycin increased the invasion inhibitory IC_50_ compared to azithromycin (10 μM). The additional removal of the desosaminyl sugar (azithromycin desglycan, IC_50_ >1,600 μM) resulted in loss of invasion inhibitory activity compared to azithromycin. Data represent the mean and SEM of three or more experiments (**P ≤0.01, ***P ≤0.001)

We tested a panel of macrolide analogues to determine whether the IC_50_ of merozoite invasion inhibition could be reduced. An erythromycin A L-megosamine sugar derivative (Meg-erythromycin, 6-O-megosaminyl erythromycin A) that has a lower IC_50_ in 1 cycle growth inhibition assays [41] was tested and found to reduce the IC_50_ of merozoite invasion inhibition (13 μM) 32-fold compared to the parent compound (420 μM; Fig. 7a; Table 1; Fig. 2b). Furthermore, addition of an oxime group (N-OH) to erythromycin A (erythromycin oxime) lowered the invasion inhibitory IC_50_ (150 μM) almost 3-fold compared to erythromycin A. The increased potency of Meg-erythromycin and erythromycin oxime compared to the parent drug indicates that various modifications to macrolides can lead to more potent invasion inhibitory activity.

Next we obtained a small panel of azithromycin analogues (in DMSO) from GlaxoSmithKline (Tres Cantos, Spain) [42–44] with reported IC_50_ values lower than azithromycin in a 72 hour growth inhibition assay (roughly equivalent to 1 cycle assays in this study; Fig. 1c). In our studies, we found that azithromycin dissolved in DMSO was less effective so the following comparisons use the IC_50_ values for both azithromycin and the azithromycin analogues with DMSO as the solubilization vehicle. Two compounds (12e, 15 μM; 1j, 7 μM) had a substantially lower IC_50_ for merozoite invasion inhibition than azithromycin (38 μM (when prepared in DMSO); Fig. 7b; Table 1; Fig. 2a), indicating that azithromycin can also be modified to lower its invasion inhibitory activity *in vitro*.

When early ring-stage parasites were treated with the compounds 1j and 12e for 40 hours (in cycle; 1j, 0.9 μM; 12e, 1 μM) and 90 hours (1 cycle; 1j, 0.7 μM; 12e, 0.6 μM) the IC_50_ of the modified analogues was between 10- and 14-fold lower than that of azithromycin (DMSO; in cycle, 12 μM; 1 cycle, 7 μM; Fig. 7c; Table 1). In contrast, parasites treated for 120 hours (2 cycle; delayed death, Fig. 1d) showed a small increase in the potency of azithromycin (DMSO, 0.1 μM) over 1j (0.16 μM) and 12e (0.16 μM) for the ‘delayed death’ inhibition typical of apicoplast ribosome targeting (Table 1). This would suggest that the apicoplast ribosome targeting is not affected by the modifications of 1j and 12e, and is a further indication that invasion inhibition or parasite growth inhibition over 1 cycle of parasite growth or less is not a result of inhibition of apicoplast ribosome activity. Further, the ability of azithromycin and its analogues to inhibit intracellular parasite growth during in cycle assays (from early rings to late trophozoites) at IC_50_ values lower than the invasion inhibition assays suggests that the compounds can inhibit parasite growth independent of merozoite invasion.

In order to assess whether the invasion inhibitory phenotype of a more potent analogue was similar to our observations for azithromycin in ethanol (see Fig. 4b), live filming of merozoite invasion in the presence of analogue 12e was examined using live video microscopy. As was observed in Fig. 4, for the no drug treatment control 53 % of merozoites that contacted an erythrocyte invaded, 29 % deformed but failed to invade the erythrocyte after contacting and 18 % released the erythrocyte membrane without invading or deforming the erythrocyte (Fig. 7d). In contrast, addition of 12e at 122 μM (2 × IC_80_) reduced invasion to only 8 % of total contact events, reduced contacts leading to erythrocyte deformation to 27 % of contact events and increased the number of merozoites releasing without invading or deforming the erythrocyte to 65 % of all contact events, resulting in failure to invade for 92 % of all contact events. These results support the live filming observations for azithromycin in ethanol (Fig. 4b) and again indicate that azithromycin acts early in the invasion process and inhibits tight junction formation. We compared the invasion phenotype of 12e to that of azithromycin in DMSO at an equal concentration (approximately 1 × IC_80_ of azithromycin in DMSO). As expected, at the same concentration the less potent parent azithromycin was less inhibitory to invasion (25 % contact events invaded) and as a result had fewer failed invasion events (75 %). Together, these findings establish a proof-of-concept that macrolides with more potent invasion inhibitory activity can be developed through chemical modification and that the dual activities of invasion inhibition and other inhibitory activities can be developed in single compounds and are not mutually exclusive.

### Identification of structural groups on azithromycin important for invasion inhibitory activity

We sought to identify structural groups of azithromycin that were most important for invasion inhibitory activity by determining the effect of removing one or both of the glycosylated groups. The two glycan groups of azithromycin, in particular the desosamine group, have been identified as critical to proper drug binding and inhibition of bacterial ribosome translation [39]. Removal of the cladinosyl group (descladinosyl) for both azithromycin and erythromycin A led to an increase of the drugs IC_50_ for both in cycle (azithromycin, 6 μM, AZR-descladinosyl, 39 μM; erythromycin A, 230 μM, ERY-descladinosyl, 288 μM) and 1 cycle (azithromycin, 7 μM; AZR-descladinosyl, 31 μM; erythromycin A, 52 μM; ERY-descladinosyl, 185 μM) drug assays (Table 1; Fig. 2a,b). Similarly, removal of the cladinosyl group from azithromycin (AZR-descladinosyl, 50 μM) led to 5-fold reduction in the invasion inhibitory activity of the compound compared to azithromycin (azithromycin, 10 μM, Fig. 7e).

We were further able to remove both the cladinosyl and desosamine groups from azithromycin (AZR-desglycan; Fig. 7e; Table 1; Fig. 2a), which led to a dramatic loss of invasion inhibitory activity and an increase in the IC_50_ beyond the limits of the assay (>1,600 μM). Comparison of these compounds indicates that the cladinosyl group has a role in lowering the IC_50_ of invasion inhibition for azithromycin, but the presence of both the cladinosyl and desosamine groups is critical for azithromycin’s invasion inhibitory activity. While these data suggest that the desosamine group plays the critical role during invasion inhibition, technical limitations prevented the selective removal of the desosamine group.

### Azithromycin inhibits *P. berghei* merozoite and *Toxoplasma gondii* tachyzoite invasion, but not *P. berghei* sporozoite invasion

**Fig. 8:**
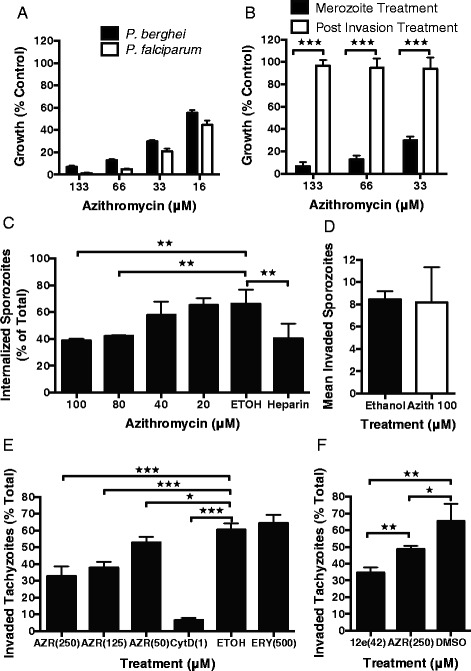
Azithromycin inhibits *P. berghei* merozoite and *Toxoplasma gondii* tachyzoite, but not sporozoite, invasion of host cells. (**a**) Azithromycin inhibited purified *P. berghei* merozoite invasion at similar concentrations compared to *P. falciparum*. (**b**) Treatment of *P. berghei*-infected erythrocytes immediately after invasion with azithromycin for 30 minutes did not result in a loss of late-stage parasites detected by flow cytometry, confirming that azithromycin inhibited invasion and not parasite growth. (**c**) Azithromycin was found to have a small but significant effect on the number of *P. berghei* sporozoites that had entered or traversed host cells. However, using a more specific assay that measures successfully invaded hepatocytes containing a developing parasite (**d**), there was no significant inhibition in sporozoites that had invaded and formed a parasitophorous vacuole. (**e**) Azithromycin treatment of *T. gondii* tachyzoites at concentrations of 250, 125 and 50 μM resulted in a dose-dependent inhibition of host cell invasion. The invasion inhibitory control cytochalasin D (1 μM) was a considerably more potent inhibitor of tachyzoite invasion, while erythromycin A, as was found for *P. falciparum*, showed no evidence of invasion inhibitory activity at concentrations up to 500 μM. (**f**) Azithromycin analogue 12e (42 μM) was significantly more inhibitory to *Toxoplasma* tachyzoite invasion than azithromycin (250 μM, both solubilized in DMSO) even when tested at a 6-fold lower concentration. Data represent the mean and SEM of three or more experiments, significance of differences was tested using an unpaired t-test (*P = 0.01 to 0.05, **P ≤0.01, ***P ≤0.001)

To test whether azithromycin could inhibit invasion of *P. berghei*, widely used as a murine model of malaria, we purified merozoites [45] and allowed invasion to proceed in the presence of azithromycin. Azithromycin inhibited merozoite invasion of *P. berghei* at very similar concentrations to that seen for *P. falciparum*, with no evidence of inhibition for parallel treatments of newly invaded rings (Fig. 8a,b).

Since azithromycin was found to be an effective inhibitor of merozoite invasion, we tested whether this drug could also inhibit invasion of *P. berghei* sporozoites into liver cells *in vitro*. The effects of azithromycin on *P. berghei in vitro* liver-stage infections following invasion have been previously reported to be limited to retardation of apicoplast development with no effect on overall parasite growth (up to 65 hours) [46]; the effect of azithromycin on sporozoite invasion has not directly been examined. Initially, we assayed the ability of *P. berghei* sporozoites to enter liver cells *in vitro* using an established assay. GFP-expressing sporozoites were allowed to settle on and invade HepG2 cells. After free sporozoites were removed by washing, cells were fixed and then antibody-labeled without cell permeabilization, thereby preventing labeling of sporozoites that are inside host cells. We then compared the number of labeled cells (external) to the total number of cells (internal and external, as measured by GFP expression) to determine invasion efficiency. There was a statistically significant, but modest, effect indicating that azithromycin may inhibit sporozoite invasion of hepatocytes at concentrations up to 100 μM azithromycin (100 μM, 42 % reduction in cell entry; (azithromycin prepared in ethanol); Fig. 8c). However, this assay is unable to robustly distinguish between sporozoites traversing liver cells from those establishing an infection. Therefore, to assess the impact of azithromycin treatment on the establishment of successful infection of liver cells, we repeated the treatment and then allowed any successfully invaded sporozoites to develop for 24 hours; this is a point at which parasites developing within the liver cell can be distinguished from any remaining sporozoites that have not established infection. The number of parasites successfully establishing an infection and forming a vacuole at 24 hours did not differ between azithromycin-treated and control (ethanol-treated) cultures. This suggests that while there was some evidence of reduced sporozoite invasion of liver cells, this did not result in measurably reduced hepatocyte infections by *P. berghei* sporozoites (Fig. 8d).

We next explored whether azithromycin could inhibit invasion of the related apicomplexan parasite, *Toxoplasma gondii*, which has orthologues of many *Plasmodium* invasion proteins and a similar invasion process [47]. There was a significant dose-dependent inhibition of tachyzoite invasion at azithromycin concentrations (in ethanol) between 250 μM and 50 μM (Fig. 8e). Azithromycin inhibited 47 % of tachyzoite invasions at the highest concentration tested (250 μM) compared to 90 % inhibition for the invasion inhibitory control cytochalasin D (1 μM) [48] and the invasion inhibitory IC_80_ of azithromycin against *P. falciparum* merozoite (38 μM; in ethanol), clearly indicating that azithromycin is a less potent inhibitor of *T. gondii* invasion *in vitro*. Importantly, there was no evidence of tachyzoite invasion inhibition in the presence of 500 μM erythromycin A, reflecting the results of the *P. falciparum* studies which showed that erythromycin A is a poor inhibitor of merozoite invasion compared to azithromycin. We next explored whether the azithromycin analogue 12e, which was found to be a more potent inhibitor of *P. falciparum* merozoite invasion than azithromycin (DMSO as vehicle), had improved tachyzoite invasion inhibitory activity compared to azithromycin. Analogue 12e (tested at 42 μM due to limited compound availability) was found to be a consistently more potent inhibitor of *T. gondii* tachyzoite invasion (48 % inhibition compared to non-inhibitory control) than much higher concentrations of azithromycin (250 μM, 24 % inhibition, Fig. 8e), suggesting that analogues of azithromycin could be developed to have much greater potency against *T. gondii* host cell invasion than the parent compound. These data suggest that azithromycin and analogues are effective inhibitors of *Plasmodium* spp. merozoite and *T. gondii* tachyzoite invasion, raising the possibility that azithromycin may target proteins or events conserved between organisms. However, there are clearly differences in drug potency between parasites hence drugs will have to be optimized to target invasion for each organism.

## Discussion

Successful control and treatment strategies for malaria currently rely on the availability of effective antimalarial drugs. The emergence of resistance to the critically important artemisinin family of drugs [7–9] highlights the need for new drugs to partner and replace current therapies, and new drug development strategies. Drugs that target merozoite invasion have been proposed as a novel strategy for antimalarial development [11–14]. We have applied robust methods for the purification of viable *P. falciparum* merozoites and invasion assays [13, 18] to screen for inhibitors of merozoite invasion. The antibiotic azithromycin and related compounds were identified as rapid inhibitors of *P. falciparum* merozoite invasion into erythrocytes. This is the first time that an antimalarial in clinical use has been linked to inhibition of merozoite invasion *in vitro*, and the first clear identification of antimalarial compounds with dual mechanisms of action against merozoite invasion and intra-erythrocytic parasite development. Live video microscopy revealed that azithromycin acts to prevent the essential step of tight junction formation during invasion. Furthermore, the inhibitory activity of azithromycin was largely ablated by selective removal of glycan groups, and we identified modified macrolides with increased potency against merozoite invasion.

Azithromycin is a macrolide antibiotic with a 15-membered macrolactone ring that is well tolerated and safe for clinical use by children and pregnant women [28]. Azithromycin and related macrolides are known to inhibit ribosomal protein synthesis in the asexual-stage parasite apicoplast by binding to the 50S subunit of the apicoplast (70S) ribosomal complex [49, 50]. Typically, clinical concentrations of azithromycin based on current dosing regimens result in ‘delayed death’ of the parasite in *in vitro* assays. The progeny of azithromycin-treated parasites fail to form functional apicoplasts in the daughter merozoites, thereby causing the death of the second generation of parasites after treatment [31, 32]. Azithromycin has been considered a candidate for inclusion in artemisinin combination therapies (ACT) and for the prevention of malaria in pregnancy [51] due to its safety and its long half-life (over 50 hours) [25, 27, 29].

In addition to its ‘delayed death’ effect on parasite growth, azithromycin has been reported to kill parasites within the first cycle of *in vitro* parasite growth at a drug concentration just above reported peak plasma concentrations [29, 32]. It has been speculated that inhibition of parasite growth during the first cycle of treatment is a result of azithromycin having a secondary target in addition to the apicoplast ribosome [32, 41]. Given growth inhibition via apicoplast-targeting ‘delayed death’ is approximately 150-fold more potent than that achieved for 1 cycle growth inhibition *in vitro*, azithromycin is thought to act predominantly through targeting the apicoplast when used clinically. Because of azithromycin’s safety profile, several studies have investigated modifications to the drug with a view to lowering the IC_50_ of short-term treatment (1 cycle of growth) to the low nanomolar range [42–44]. Yet despite this interest in azithromycin, the target of drug inhibition during the first cycle of growth is unknown, and the potential of this novel mechanism of action as a clinical treatment remains unclear.

Several lines of evidence indicate that azithromycin has a secondary mechanism and target(s) of action to inhibit invasion, in addition to binding to the 50S apicoplast ribosomal subunit as described for the ‘delayed death’ phenotype [31, 32]. Clindamycin, a smaller and structurally unrelated antibiotic that has overlapping binding sites to azithromycin on the apicoplast ribosome [39], as well as an identical mode of action, had very little invasion inhibitory activity. Selection of a D10 line for resistance (57-fold reduced sensitivity) to azithromycin in the 2 cycle ‘delayed death’ assays did not result in a similar increase in resistance to azithromycin invasion inhibition assays when compared to a parental D10 (‘delayed death’ sensitive) line. Comparison of azithromycin and its modified analogues (1j, 12e) showed that modifications increasing the potency of these analogues over azithromycin in invasion inhibition, in cycle (40 hour) and 1 cycle (90 hour) assays did not appreciably increase the potency of the compounds over 2 cycle ‘delayed death’ assays, suggesting the mechanism of enhanced inhibitory activity was independent of anti-ribosomal activity.

The near instantaneous inhibition of merozoite invasion caused by azithromycin also suggests that this drug can rapidly kill parasites through a secondary mechanism of action. Typical ‘delayed death’ inhibition by macrolide antibiotics results in the loss of apicoplast functionality [31, 32], with this then leading to the loss of isoprenoid precursor biosynthesis, the only essential function of the apicoplast during blood-stage development [33]. The long timeframes required for apicoplast translation inhibition and the resulting ‘delayed death’ caused by macrolide antibiotics is in striking contrast to the very rapid inhibition of merozoite invasion at higher drug concentrations. Furthermore, removal of the apicoplast in the experiments by Yeh et al. [33] was not reported to result in an invasion defect. Combined, the available evidence suggests it is very unlikely that the apicoplast plays some as yet undefined role in merozoite invasion and implicates a secondary mechanism of action of azithromycin against invading merozoites. Whether the mechanism of invasion inhibition and intracellular asexual-stage parasite growth inhibition (within 1 cycle in the absence of invasion inhibition) is the same remains to be elucidated. However, the similar IC_50_ values seen for both inhibitory phenotypes raises the intriguing possibility that this secondary mechanism of action could be targeted throughout the disease causing blood-stage of *P. falciparum* malaria.

The ability of azithromycin to rapidly inhibit merozoite invasion, in addition to the apicoplast-targeting ‘delayed death’ mechanism of action, is an exciting prospect for the development of macrolide-based drugs in combination therapies. This dual modality could see azithromycin assist in rapid clearance of parasites as well as offering longer-term treatment of the remaining low-level blood and liver-stage parasites through its activity against the apicoplast. Such a drug treatment strategy has the potential to increase drug efficacy, while reducing the chances for the development of resistance.

Although the *in vitro* merozoite invasion inhibitory activity of azithromycin required drug concentrations at the upper end of what is achieved clinically [29, 32], we demonstrate that modification of the macrolides can lead to a substantial improvement in invasion inhibitory activity; this suggests that invasion inhibitory macrolides could be developed as therapeutics. Addition of an L-megosamine sugar to erythromycin A through an *in vivo* fermentation process [41] produced a compound with 32-fold greater invasion inhibitory activity than parent erythromycin A. Furthermore, a selection of azithromycin analogues [42–44] lowered the invasion inhibitory IC_50_ by up to 5-fold. The azithromycin derivatives were tested only when solubilized in DMSO due to limitations in the amount of available material. Importantly, the use of DMSO as vehicle for solubilization reduced the potency of the parent compound azithromycin 4-fold relative to ethanol as vehicle. If this observation were to hold true for the derivatives, the invasion inhibitory IC_50_ of the compound 1j (5) [44] would be in the range of 1–2 μM.

The invasion inhibitory IC_50_ of azithromycin (10 μM) and analogues (1j, 7 μM; 12e, 15 μM) is comparable to that achieved for inhibitors that specifically target the function of essential merozoite proteins. Two recent proof-of-concept studies identified small molecule invasion inhibitory compounds targeting interactions between essential invasion ligands RON4-AMA1 (inhibitory range 30–6 μM) and MSP1-19 (replication inhibition 21.7 μM) [12, 14], and reported invasion inhibitory IC_50_ values in a very similar range to azithromycin and analogues. Another highly selective invasion inhibitor, the 3D7-AMA1-specific peptide inhibitor R1 [52], has an invasion inhibitory IC_50_ of 2.5 μM in assays using purified merozoites [18]. Azithromycin, with minimal modification, can rapidly inhibit merozoite invasion at concentrations comparable to some of the best and most selective invasion inhibitors available for drug development and research. The encouraging improvements evident in the invasion inhibitory activity of azithromycin from only a small panel of derivatives, combined with the proven safety and efficacy of macrolides, support the potential for developing such compounds as therapeutics.

Removal of azithromycin’s glycan groups provided insight into the structural requirements for invasion inhibitory activity. Removal of the cladinosyl sugar resulted in a 5-fold reduction in invasion inhibitory activity. Strikingly, removal of both sugars resulted in complete loss of invasion inhibitory activity, strongly suggesting that the desosamine sugar, rather than the cladinosyl, is critical for invasion inhibitory activity. Interestingly, the desosamine sugar is also considered the critical glycosylated group in binding of macrolides to microbial ribosomes [39] with clinically used macrolide antibiotics such as telithromycin having dispensed with the cladinosyl group altogether. Screening of a larger number of macrolides and modified analogues will help identify others with greater potency, and allow assessment of which modifications and groups are most important for activity.

The fact that azithromycin inhibits host invasion by *Plasmodium* merozoites (from human and rodent malaria) and *T. gondii* tachyzoites parasites suggests that the target of inhibition is shared amongst some apicomplexan invasive stages. The fact that the potency of azithromycin against *P. falciparum* (human) and *P. berghei* (rodent) malarias was very similar raises the possibility that targeting this pathway could be an effective strategy to treat other malaria species. The results of the *T. gondii* invasion assays suggest that host cell invasion of this more distantly related apicomplexan parasite can also be targeted by azithromycin, albeit at higher concentrations. Importantly, the analogue 12e had equal or better invasion inhibitory potency than azithromycin (in ethanol or DMSO, respectively) when tested at a 6-fold lower concentration. This strongly suggests that analogues of azithromycin could be developed with superior inhibitory activity against tachyzoite invasion and confirms that the invasion inhibitory activity of azithromycin and analogues is shared across distantly related apicomplexan parasites.

Macrolide antibiotics have been linked to mechanisms of action other than binding to microbial ribosomes, including interference with intracellular signalling mechanisms such as Ca^2+^ and MAPK through an as yet undefined mechanism (reviewed in [53]). Intracellular Ca^2+^ signalling is thought to play an important role in invasion (reviewed in [54]) so interference with this mechanism by macrolide antibiotics could contribute to inhibition of invasion. Macrolide antibiotics have also been shown to bind to negatively charged phospholipid bilayers and interfere with normal membrane function [55, 56]. Intriguingly, the relative activity of the macrolides appeared to be in part related to the number of cationic groups present on the macrolide.

## Conclusions

The results of this study have identified for the first time that macrolide antibiotics, a diverse group of well-tolerated antibiotic drugs with antimalarial properties, inhibit *Plasmodium* spp. merozoite invasion into erythrocytes *in vitro*. Invasion inhibition assays and live filming experiments show that azithromycin acts rapidly to stop merozoite entry very early during the invasion process. The mechanism of invasion inhibition appears to be independent of apicoplast ribosomal translation inhibition, which is the currently described mechanism of action of these drugs. Modification of both azithromycin and erythromycin A resulted in promising reductions in the invasion inhibitory IC_50_ to levels nearing those achieved clinically for the parent compounds, providing a proof-of-concept for the potential development of potent invasion inhibitors. Elucidation of the mechanism of action and subsequent improvements in compound potency could pave the way for development of macrolide antibiotics with dual modality as novel and potent antimalarials to complement frontline treatments for malaria. Furthermore, the use of drugs that inhibit merozoite invasion in combination with drugs that inhibit intra-erythrocytic parasite development may be an effective strategy to facilitate rapid parasite clearance to help improve clinical outcomes, reduce transmission, and minimize the development of resistance.

## Methods

### Culture of *P. falciparum*

D10 parental and GFP fluorescent D10-PfPHG [57] parasites were cultured in human O^+^ erythrocytes (Australian Red Cross, Victoria, Australia) according to established protocols [57, 58]. Synchronization using heparin for growth inhibition and merozoite invasion inhibition assays has been described previously [13, 18, 23, 57]. Unless otherwise stated, the D10-PfPHG isolate was used in assays because the GFP label facilitates accurate quantification of parasite stages by flow cytometry [13, 18].

### Drug inhibition assays

A diagram outlining the different drug inhibition assays used in this study to measure *P. falciparum* growth and invasion inhibition is available in Fig. 1a,b,c,d,e. The protocol for measuring drug inhibition of ring- and trophozoite-treated parasites in cycle (approximately 40 hours post-invasion), 1 cycle (90 hours post-invasion, includes 1 cycle of replication) and 2 cycle (120 hour post invasion, includes 2 cycles of replication) growth assays has been described in detail previously [13, 57, 59]. Parasitaemia was measured at trophozoite stage by flow cytometry (BD FACSCalibur, BD Biosciences, Franklin Lakes, NJ, USA) after staining with 10 μg/ml ethidium bromide (EtBr) for 1 hour prior to washing in PBS. Typically, 20,000 to 40,000 erythrocytes were counted for each well. Samples were analyzed using FlowJo software (Tree Star Inc, Ashland, OR, USA).

### Invasion inhibition assays

The protocols for filtration of viable *P. falciparum* and *P. berghei* merozoites from E64-treated schizonts and merozoite invasion inhibition assays have been previously described [13, 18, 45]. Parasitaemia was assessed by flow cytometry of EtBr-treated rings (1 hour post-invasion, 5 μg/ml EtBr, staining 10 minutes, no wash) and trophozoite stages (as per 1 cycle assay). Early ring-stage parasites were gated using a Fl-1 high (GFP) and Fl-2 low (EtBr) gate. Trophozoites were gated using a Fl-1 high (GFP) and Fl-2 high (EtBr) gate. Parasitized erythrocytes for microscopy were fixed in methanol and stained for 10 minutes in 10 % Giemsa (Merck, Kenilworth, NJ, USA). For microscopy of fixed slides, the number of early ring-stage parasites in a minimum of 1,000 RBCs was assessed by an experienced microscopist in duplicate wells from three independent experiments.

Liver-stage assays used *P. berghei* ANKA sporozoites dissected from infected *Anopheles stephensi* mosquitoes. Two-color invasion assays followed the protocol of Sinnis et al. [60] with the following modification. *P. berghei* constitutively expressing GFP [61] were added to cultured HepG2 cells in the presence of inhibitory compounds, centrifuged for 3 minutes at 150 g and then incubated for 1 hour to allow invasion. The liver cell and parasites were then fixed but not permeabilized. When probed with polyclonal antibody against *P. berghei* merozoite surface proteins [62] (500:1) followed by anti-mouse Alexa Fluor 546 (1,000:1; Invitrogen, Carlsbad, CA, USA) only uninvaded sporozoites are labelled. Without permeabilization, the antibodies cannot pass through the HepG2 cell plasma membrane, so sporozoites within the liver cells are not labelled. Invasion rate is calculated by comparing the number of labelled sporozoites to the total number based on GFP expression. Successful infection of hepatocytes was measured through an extension of the invasion assay by adding the parasites as above, incubating at 37 °C for 2 hours, exchanging media and then incubating the liver-stage parasites for a further 24 hours to allow development of any intracellular parasites. The presence of developing liver-stage parasites that had successfully invaded and established infection was then quantified according to previously published methods [63]*.*

*Toxoplasma gondii* invasion assays were performed using the Rh parasite strain according to existing protocols [64] with the following modification. Inhibitory compounds were added to both the Endo and invasion buffers prior to commencement of host cell invasion. Parasites were allowed to invade for 15 minutes, fixed and sequentially labeled with αSAG1 (2,000:1) and αGAP45 antibodies (500:1), followed by secondary labeling with Alexa Fluor 488/543 (Invitrogen). Parasites were imaged with a Leica SP2 confocal microscope (Leica, WETZLAR, Germany) and a minimum of 1,000 parasites were counted per well with three replicates for each concentration.

The IC_50_ and IC_80_ concentrations were determined using GraphPad Prism (GraphPad Software, La Jolla, CA, USA) as previously described [13]. The inhibitory concentration (IC) for 1 cycle (90 hour), 2 cycle (‘delayed death’, 120 hour) and merozoite (<1 hour post-invasion) will be discriminated by the addition of ^*90hr*^, ^*120hr*^ and ^*mero*^ where appropriate.

### Live cell microscopy

Live cell microscopy experiments were undertaken according to the method of Weiss et al. [38]. Briefly, highly synchronous late-stage schizonts were diluted 1:25 in media ± drug treatment and allowed to settle to produce a monolayer onto a 35 mm FluoroDish (World Precision Instruments, Sarasota, FL, USA). At 37 °C on a Zeiss Axio Observer.Z1 fluorescence microscope (Zeiss, Oberkochen, Germany) equipped with humidified gas chamber (90 % N_2_, 5 % O_2_ and 5 % CO_2_), selected schizonts were observed until they ruptured and released their merozoites. Time-lapse videos of the invading merozoites were recorded with a high-resolution AxioCam MRm camera (Zeiss). ImageJ was used to perform image analysis and GraphPad Prism used to perform statistical analyses using a Chi-squared test.

### Chemistry

A summary of the chemical structures of the compounds used in this study is available in Fig. 2. Azithromycin 1, erythromycin 9, erythromycin oxime 11 and dirithromycin 13 were obtained commercially from Ak-Scientific (Union City, CA, USA). Roxithromycin 12, clindamycin 16 and spiramycin 15 were from Sigma Aldrich (St Louis, MO, USA). 6-O-Megosaminyl erythromycin A 14 was a gift from C Goodman, E Rodriguez and H Gramajo [41]. Compounds 4–8 were provided by GlaxoSmithKline (Tres Cantos, Spain). Synthesis of these compounds are as previously described: 4 (12e) and 5 (1j) [44]; 6 (11c) [42]; and 7 (7/47) and 8 (7/25) [43]. AZR-desclad 2 was prepared according to Istuk et al. [65]. ERY-desclad 10 was prepared following methods of LeMahieu et al. [66]. AZR-desglycan 3 was prepared following similar procedures previously outlined in the literature [67, 68]. In general, compounds were solubilized with ethanol as vehicle, with the exception of the initial characterization of azithromycin (1), where both ethanol and DMSO were compared, and compounds 4–8 (DMSO), owing to the limited amount of material available.

### Availability of supporting data

A tabular summary of data used to generate graphs and perform statistical tests presented in this manuscript (Figs. 3b,c; 4; 5e; 6a; 7d; and 8b,e,f) is available in Additional file 3.

## Additional files

Additional file 1: Video S1. Video of newly egressed merozoites revealed that of those that contact erythrocytes about half progress to invasion (green arrows), whilst the remainder either detach after contact (red arrow) or progress to deformation but fail to invade (blue arrow). Additional file 2: Video S2. Video of newly egressed merozoites from schizonts in the presence of azithromycin 134 μM revealed that azithromycin inhibits early events in invasion; merozoites could attach to erythrocytes but not invade (red arrows) and did not show evidence of tight junction formation. Additional file 3:
**Supporting data.**

## Abbreviations

DMSO: Dimethyl sulfoxide
EtBr: Ethidium bromide
GFP: Green fluorescent protein
RBC: Red blood cell
SEM: Standard error of the mean
WHO: World Health Organization
