# Supplementary material for: Macrolides rapidly inhibit red blood cell invasion by the human malaria parasite, Plasmodium falciparum
Source: BMC Biol. 2015 Jul 18;13:52. doi: 10.1186/s12915-015-0162-0 (PMC4506589; doi:10.1186/s12915-015-0162-0)
Supplement: Additional file 3: — Supporting data. [file 12915_2015_162_MOESM3_ESM.docx]

**Wilson *et al*. Additional File 3: Supporting Data.**

**Supporting Data 1: Tabular information for** **Figure 3B**

| **Treatment (μM)** | **Treatment Stage** | **n=** | **Mean Growth** | **± SEM** |
| --- | --- | --- | --- | --- |
| **Azithromycin (151)** | RBC Pretreatment | 6 | 88.66 | 5.101 |
|  | T=0 | 6 | 1.42 | 0.627 |
|  | T=20 | 6 | 78.88 | 8.769 |
| **Heparin** | RBC Pretreatment | 3 | 114.8 | 3.229 |
|  | T=0 | 3 | 4.06 | 2.349 |
|  | T=20 | 3 | 85.16 | 11.73 |

**Supporting Data 2: Tabular information for** **Figure 3C**

| **Treatment (μM)** | **Treatment Stage** | **n=** | **Mean Growth** | **± SEM** |
| --- | --- | --- | --- | --- |
| **Azithromycin (380)** | RBC Pretreatment | 5 | 81.73 | 3.049 |
|  | Merozoite | 5 | 9.688 | 0.889 |

**Supporting Data 3: Tabular information for** **Figure 4**

| **Treatment (μM)** | **Contact events** | **Chi-squared** | **df** |
| --- | --- | --- | --- |
| **No Drug vs Azr (56)** | 22 vs 28 | 1.70 | 2 |
| **No Drug vs Azr(100)** | 22 vs 16 | 16.84 | 2 |
| **Azr (56) vs Azr (100)** | 28 vs 16 | 15.73 | 2 |

**Supporting Data 4: Tabular information for Figure 5E**

| **Treatment (μM)** | **Treatment Stage** | **n=** | **Mean Growth** | **± SEM** |
| --- | --- | --- | --- | --- |
| **Roxithromycin (337)** | RBC Pretreatment | 4 | 86.46 | 11.32 |
|  | Merozoite | 4 | 23.69 | 6.8 |
| **Spiramycin (492)** | RBC Pretreatment | 3 | 65.2 | 8.925 |
|  | Merozoite | 3 | 4.75 | 0.725 |
| **Erythromycin (1682)** | RBC Pretreatment | 5 | 94.68 | 14.88 |
|  | Merozoite | 5 | 6.47 | 1.621 |

**Supporting Data 5: Tabular information for Figure 6A**

| **Treatment (μM)** | **Treatment Stage** | **n=** | **Mean Growth** | **± SEM** |
| --- | --- | --- | --- | --- |
| **Azithromycin (38)** | RBC Pretreatment | 5 | 89.75 | 13.51 |
|  | Merozoite | 5 | 27.19 | 6.976 |
| **Clindamycin (2972)** | RBC Pretreatment | 5 | 35.81 | 10.68 |
|  | Merozoite | 4 | 13.6 | 8.881 |

**Supporting Data 6: Tabular information for Figure 7D**

| **Treatment (μM)** | **Contact events** | **Chi-squared** | **df** |
| --- | --- | --- | --- |
| **No Drug vs Azr (122)** | 38 vs 98 | 9.95 | 2 |
| **No Drug vs 12e(122)** | 38 vs 114 | 41.49 | 2 |
| **Azr vs 12e** | 98 vs 114 | 25.31 | 2 |

**Supporting Data 7: Tabular information for** **Figure 8B**

| **Treatment (μM)** | **Treatment Stage** | **n=** | **Mean Growth** | **± SEM** |
| --- | --- | --- | --- | --- |
| **Azithromycin (133)** | Merozoite | 3 | 6.812 | 2.252 |
|  | Post-Invasion | 3 | 96.83 | 1.783 |
| **Azithromycin (66)** | Merozoite | 3 | 12.88 | 1.681 |
|  | Post-Invasion | 3 | 94.73 | 4.829 |
| **Azithromycin (33)** | Merozoite | 3 | 29.72 | 2.02 |
|  | Post-Invasion | 3 | 93.99 | 5.051 |

**Supporting Data 8: Tabular information for** **Figure 8C**

| **Treatment (μM)** | **n=** | **Mean Internalized** | **± SEM** |
| --- | --- | --- | --- |
| **Azithromycin (100)** | 3 | 38.92 | 0.8085 |
| **Azithromycin (80)** | 4 | 42.24 | 0.1783 |
| **Azithromycin (40)** | 4 | 57.76 | 5.021 |
| **Azithromycin (20)** | 4 | 65.33 | 2.547 |
| **Ethanol** | 5 | 66.27 | 4.703 |
| **Heparin** | 4 | 40.53 | 5.391 |

**Supporting Data 9: Tabular information for** **Figure 8D**

| **Treatment (μM)** | **n=** | **Mean Invasion** | **± SEM** |
| --- | --- | --- | --- |
| **Ethanol** | 3 | 8.45 | 0.4359 |
| **Azithromycin (125)** | 3 | 8.197 | 1.824 |

**Supporting Data 10: Tabular information for** **Figure 8E**

| **Treatment (μM)** | **n=** | **Mean Invasion** | **± SEM** |
| --- | --- | --- | --- |
| **Azithromycin (250)** | 4 | 32.67 | 2.974 |
| **Azithromycin (125)** | 4 | 37.80 | 1.78 |
| **Azithromycin (50)** | 3 | 52.81 | 2.051 |
| **Cytocholasian D (1)** | 3 | 6.333 | 0.8819 |
| **Ethanol** | 5 | 60.47 | 1.665 |
| **Erythromycin (500)** | 4 | 64.33 | 2.522 |

**Supporting Data 11: Tabular information for** **Figure 8F**

| **Treatment (μM)** | **n=** | **Mean Invasion** | **± SEM** |
| --- | --- | --- | --- |
| **12e (42)** | 3 | 34.65 | 1.774 |
| **Azithromycin (250)** | 3 | 48.67 | 1.202 |
| **DMSO** | 3 | 65.50 | 5.172 |
